# Remarks and Observations on the Epidemic Catarrh

**Published:** 1818

**Authors:** James Carver

**Affiliations:** Veterinary Surgeon


### FACTS,

INTERESTING TO THE

### *Gentleman, the Farmer,*

### THE GRAZIER AND THE BUTCHER;

BEING AN ACCOUNT OF AN

### Epidemic Disease,

Which has lately been prevalent in England, and now prevailing in this city, and on the Lancaster line of stages, belonging to John Tomlinson and others;

TOGETHER WITH

### *The Causes, Symptoms, and best mode of Treatment,*

BY WHICH NUMBERS HAVE BEEN SAVED.

BY DR. JAMES CARVER,
VETERINARY SURGEON.

ALSO, AN ACCOUNT OF THE SAME DISEASE AMONG

### OXEN, COWS AND SHEEP.

### REMARKS AND OBSERVATIONS

ON THE

### EPIDEMIC CATARRH,

Which prevailed in this city during the winters of 1815, 16, 17 and 18, and various other parts in and surrounding the city of Philadelphia, and now prevailing on the Lancaster line.

IT should be understood that the epidemical diseases of horses generally assume the form of catarrh or cold, affecting principally the membranes of the nose, throat, windpipe and lungs, and are often accompanied with fever. The disease varies considerably in degree; sometimes attacking with great violence, at others so inconsiderable as to require only an opening diet, and a few doses of some cooling medicine for its removal. This distinction, however, requires the judgment and experience of a veterinary physician.

In the winter and spring of 1814, this disease was very prevalent at the Veterinary College, during my studies at that institution under Professor Coleman.

Osmer, in his treatise on the diseases of horses, has given a good description of an epidemic that raged among horses in the year 1750. Some, he observes, had a discharge of matter from the eyes, nose and mouth; others had none; but in all, there were tokens of inflammation, attended with fever and violent cough. Most of the horses, which had a discharge from the nose, &c. lived; and where such discharge did not happen, or a critical abscess fell on some part, most of them died. He was led to try the use of rowels, and soon experienced a good effect from them; the horse in about thirty hours became more cheerful, and recovering his appetite, and in another day, generally appearing quite well. Rowels had the same good effect in horses that discharged at the nose, and they got over the disease much sooner than those who had no such assistance. If the horse be attacked with violent fever, and dry cough, without any discharge from the nose, he advises being bled largely, but observes that whenever there was a discharge from the nose, bleeding appeared to do harm. In both circumstances, however, he advises cooling medicines, and clysters occasionally.

A stud of mares and colts, of various ages, were attacked with this distemper: some had a discharge from the eyes, nose, and mouth; and some had swelling on the udder, shoulder, or the under jaw.

They were taken to a house, bled and rowelled; had nitre given to them, by which treatment the disease was soon got under, excepting in the sucking foals. When swellings appeared in any part, they were opened as soon as matter had formed in them. Some had setons inserted in the depending part of the swellings, with a view of drawing off the febrile matter, but this treatment proved unsuccessful; the tumour neither advanced nor receded. The setons were then taken out, and no medicines given, and in a few days the swellings came to good matter, which being let out, the animal soon got well. But for the sucking foals no remedy availed; the disease baffled all the attempts of nature and art. If you bleed them, a swelling perhaps comes on the parts, and would remain indurate for several months; hard swellings would also arise on other parts: in short, as long as they sucked the mare the disease continued, but upon being weaned they soon got well. He observes, that the distemper began in general with considerable weakness of the limbs, so that a horse would be seen to reel and stagger, when led from the stable; there was generally a dry cough, the eyes would suddenly become dim, glared as lifeless, and the horse showed no inclination to drink.

Osmer divides the distemper into five different classes or degrees: in the first, besides the symptoms first mentioned, there was a coldness of the external parts, and the weakness was particularly observable in the hind limbs. There was also appearance of fever or inflammation.

In this class of the distemper, bleeding uniformly did harm; and if done largely, it generally proved fatal; even when the horse was costive, no advantage was derived from clysters, and rowels appeared to do harm. He advises the following medicines to be given three times a day for a few days, or until the urinary secretions appear to be augmented, and the horse appears to drink freely, upon which he generally becomes well on a sudden, recovers his limbs and his appetite, and is free from all complaint but his cough, which perhaps leaves him not entirely until he recovers his flesh. The horse's diet at first should be hay and scalded bran; his drink should be moderately warm, and given freely.

| | |
|---|---|
| Rx.—Crude sal ammoniac - | one ounce. |
| Nitre - - - | one ounce. |
| Castle Soap - - | half an ounce. |
| Camphor - - - | two drachms. |

Mx.—For one dose.

He particularly directs that this medicine be discontinued so soon as the horse appears to be relieved, and the evacuation of the urine much increased. He also advises the horse to be allowed a more nourishing diet, such as bran and oats scalded together.

In the second class, there is considerable fever; the external parts, hot and burning, and the horse appears to be affected in his head and sight.

Here he recommends moderate bleeding and clysters, as well as the medicines directed in the first class. If the fever continues twelve hours, and the membranes about the eyes appear inflamed, he thinks another moderate bleeding proper, which will generally be sufficient; but in this or future bleedings, the direction for so doing is to be solely taken from the tokens of inflammation; remembering, that in this disease, horses can bear only the loss of a small quantity at one time. The blood of horses, labouring under these symptoms, is generally very sizy; in which case he considers rowels very improper. For such as are affected with soreness of the throat, (third class) bleeding, clysters and rowels, he says are all improper, unless there be manifest tokens of fever and inflammation: in either case, the medicine before directed is proper. These horses will eat bread—water gruel, if made thin. Soreness of the throat is indicated by a difficulty in swallowing. It is seldom accompanied with much fever, and their appetite for eating and drinking seems better than in those of the first and second class; but they are in general miserably reduced before the soreness goes off, which he does not consider as the effects, solely, of their fasting.

Fourthly. Others are seized at first, with a cough only, and show little or no symptoms of illness. These, in general, have soon a discharge from the nostrils; they recover sooner than any, and frequently without assistance. He advises, however, rowels and a diet of scalded bran.

Fifthly. In others again, an abscess or boil appears soon after the cough, in some part of the head or body, which sometimes soon comes to matter. In others, their lives are manifestly endangered before an imposthumation can be obtained, even with the assistance of art. In this class of the distemper, he advises the application of poultices to the swelling, in order to bring it to matter as speedily as possible, and the medicine as before directed in the first class, &c. By these different methods, he says, he has saved the lives of many horses, having lost a few only out of a great number. But he acknowledges, that when the distemper first appeared, he endangered the lives of many.

Vegetius describes an epidemic, or what is considered a contagious fever, which corresponds nearly with Osmer, only he divides it into two classes: he recommends oily injections into the nostrils, anointing the head with oil, and keeping it warm, and giving a mixture of *diapente*, being gradually increased to two spoonfuls. He recommends also, something similar to rowels. It will appear, no doubt, to the attentive reader, that Osmer's description has the appearance of being the result of careful observation. There is also a degree of candour in his confessing, that previously to his having attentively studied the nature of the distemper, he endangered the lives of many horses, which certainly deserves praise. With respect to the description of Vegetius, though some may value it for its antiquity, it will probably be thought by most persons, that his prescribing a mixture of old wine and diapente, was by no means judicious, particularly if the operation of mixing these ingredients was left to the groom, who, possibly, might be tempted to drink the one and throw the other away.

To discover and account for the nature of the distemper among horned cattle, which raged so many years in Europe, and moreover to discover a cure, appeared at that time as difficult and unpromising a task, as it was of old, to untie the Gordian knot. From accounts of Rammazani and Lancisi, two learned physicians of those times, it raged with great violence, as appears from accounts published by them, in 1711 and 1715, which is now more than one hundred years ago.

Ancient history, both sacred and profane, furnishes us with accounts of pestilential diseases among horned cattle; and one of the sorest plagues which God inflicted on the obdurate kings of Egypt, near 1600 years before Christ, and which proved the destruction of all Egyptian horned cattle at those times. The severest plague of this kind appeared in the year 810, which not only destroyed every head of cattle in the army of the emperor Charlemagne, but through his dominions also. In 1713, in the kingdom of Naples, it extended through the country from Rome to Rome, and from hence into Germany, France, Flanders, Holland, Great Britain and Denmark.

The same countries which breed the plague and small pox, seem to have propagated this contagion. The autumnal heats in different parts of Asia and in Africa, the putrid effluvia from the Ganges and the Nile, or from corrupted stagnating waters, which contaminate the blood and juices of the cattle. In the year 1800, while residing in the district of Rungpoore, not far from the Naupal mountains, I was an eyewitness to one of those epidemics, which at that time was sweeping off the cattle by thousands, particularly the sheep, bred in those parts for the Cashmere shawl factories. Many flocks of these sheep, then near the factory where I resided, suffering very much from the severe inundation of the waters of the Ganges, which at that time had overflowed the whole country for thousands of miles, I was applied to by the natives for relief and assistance, and having on many former occasions saved many of their cattle from the various complaints with which at different times they were afflicted, I have the pleasing satisfaction of declaring, that I saved many hundreds of their flocks by inoculation with vaccine virus; an account of which I transmitted to Washington Custis, Esq. of the Arlington Society, near the seat of Washington, prior to my departure for England, and which that gentleman published in a small pamphlet, which I left in the hands of Reuben Haines, when I left this city for my studies at the Veterinary College.

The most serious epidemic that has occurred for several years to horses, made its appearance during my residence in England, in the spring of 1814, and during the months of May and June raged with great violence. In many places the symptoms were alarming, and much inconvenience was suffered from the suspension of animal labour, and the expense of veterinary medical attendance; yet it does not appear to have destroyed many horses, while in others its fatality has been considerable, but in the metropolis it is said to have made great destruction. Though much has been written on the epidemic diseases of cattle, and many conjectures suggested as to their origin or causes, it does not appear, that much light has been thrown on the subject. It must be allowed, however, that such attempts were very laudable, as they were considered the only probable means of discovering a mode of prevention, or such a mode of treating the disease, as might effectually arrest its progress, before its dreadful effects were generally felt. If we read the accounts of the authors before quoted, no doubt will remain of the contagious nature of these diseases. So destructive have they been at some periods, as to cause the most serious alarm, particularly about the beginning of the last century. According to Lancisi's account, there died in the ecclesiastical states, from October, 1713, to April 1714, 8,466 oxen used for ploughing, 10,125 white cows, 2,216 red cows, 108 breeding bulls, 427 young bulls, 451 heifers, 2362 calves, 862 buffaloes, male and female, 635 young ditto; in all, 26,252 cattle, in seven months.

This writer thinks, that if the computation had begun from the 2d of August, the number of cattle that perished would have amounted to 30,000.

It is fortunate for mankind, that so dreadful a pestilence has not happened for many years; the epidemic diseases of a much less formidable nature have several times appeared.

During my residence at the college in 1814, a great number of horses were attacked with a complaint, which Mr. Coleman called influenza, and we had at the college near twenty attacked at one time; and last winter, in this city, the same complaint made its appearance in the stable of Mr. John Carter, where six horses nearly all at one time were attacked. Mr. Carter called me in, and by following the same mode of treatment as was prescribed by professor Coleman, at the Veterinary College, I have the pleasing satisfaction to say, that every horse was saved. Mr. Carter, I recollect, was very much alarmed, and thought that that dreadful disease, the glanders, had made its appearance, and it was with great difficulty that I prevailed on him to believe the contrary.

In the latter part of the winter, seven or eight horses more, belonging to Mr. S. Allen, in Sixth-street, were all attacked with the same complaint; two of his own, and one belonging to Mr. Hampton, suffered very severely, and it was with great difficulty they were saved. Mr. Allen and also Mr. Carter will, no doubt, satisfy any inquiries that may hereafter be found necessary on this occasion.

If there is any part of my professional labours which may not have come under inquiry, or the notice of the public, here are facts in point and on the spot which will bear neither contradiction nor doubt.

An account of the symptoms and mode of treatment in this complaint, will probably not be unacceptable to my fellow citizens. The most common symptoms of this complaint was a general debility, sometimes attended with cough, and sometimes not, dullness of the eyes, attended with a small degree of inflammation, and a disinclination both for food and water. These symptoms were soon followed with a discharge from the nostrils, and soreness of the throat, and the glands about those parts causing more or less difficulty in swallowing, which the grooms complained of very much, and sometimes to such a degree, that in attempting to drink, the water would return through the nostrils, and very often some of the horse's food would be found mixed with the matter discharged from the nose. On the first appearance of the complaint, I generally found it necessary to bleed, sometimes freely, making the pulse my guide, and this I found particularly necessary when the breathing became quick and laborious, and which may always be observed by the motion of the flanks and nostrils, when the pulse was high and the inner membrane of the eye inflamed. In some of the above cases, when I found no abatement of the symptoms in ten or twelve hours, and particularly if blood had been drawn sizy and of a yellow buff colour, the bleeding was repeated; but this was not found necessary only in three instances before alluded to, at Mr. Allen's stables, and in once instance at Mr. Carter's: when the bowels were costive, I gave a mild laxative, with small doses of nitre, night and morning, and in these two cases, where no medicine was given for several days, except in a liquid form.*

* In two of the above cases, one at Mr. Allen's, and also one at Mr. Carter's stable, I found the difficulty of swallowing so great, that I contemplated the operation of bronchotomy: this operation, however, was

To relieve the soreness of the throat, blistering ointment was applied under the throat, and over the glands, as high up as the root of the ears. In one of these cases at Mr. Carter's, a rowel was inserted under the jaw, which being kept open, was found very beneficial. Blistering, however, appeared to have the best effect, though sometimes inconvenient, from the horse rubbing against the manger. When considerable weakness was observed, the patients were freely supplied with gruel or marsh, and when the throat was not sore, tonic and cordial remedies were given.

It is here necessary to observe, that tonic or cordial medicines, at the commencement of the complaint, should never be had regard to, and even when they do appear necessary at a later period of the complaint, it should always be first ascertained through the medium of the pulse, that no inflammatory symptoms exist, nor should ever be persisted in, when they appear to cause uneasiness, or diminish the appetite, or increase the frequency of the pulse. We generally find during spring, and during early summer, a catarrhal disease is very common, particularly among horses, and which has some resemblance to that just described. During the present early part of summer, a disease of that kind has been very prevalent. Mr. Roach has a fine colt, which only a few days ago had a slight attack of this complaint: this horse had been under a course of medicines, but on being removed to his own stable, where the ventilation was somewhat better than where he formerly stood, he soon recovered: though I have several patients now under treatment for the very same complaint. This complaint has attacked some horses at grass, as well as those in the stables; in fine, it has occurred under a variety of circumstances, and though young horses have appeared most obnoxious to it, many aged horses have been attacked.

not performed, though at the Veterinary College I assisted Mr. Coleman in performing this operation on eight or nine different horses for this complaint, which was attended with great relief to the animal.

And it is under these circumstances, when both old and young are attacked, apparently under the same symptoms, that it requires the judgment and experience of a veterinary physician.

The most common symptoms of this complaint is a cough with a soreness of the throat, though this symptom does not always occur at the commencement of the disorder, but often comes on during the progress of the complaint. The cough is commonly the first symptom that attracts notice, and is generally accompanied, even at first, with a loss of both spirits and appetite. A discharge from the nostrils generally takes place after three or four days, and sometimes the matter is of a whitish colour, like strangles, and which I always found in those cases at the college as well as in the present, a favourable symptom; at others, it is mixed with the horse's food, and consists in a great measure of what he endeavours, though unable, to swallow. This indicates inflammation of the throat, and requires the immediate application of a blister. In almost every case that I met with, both in this city and at the college, I found bleeding to be proper; and in some instances, where I found horses attacked with great severity, the pulse much increased, the breathing, and heaving of the flanks quicker than usual, with some inflammation of the eye, and the first drawn blood sizy, and thickly covered with a buff, I have found repeated bleeding, twice and three times, of the greatest advantage. But this should not be done without great caution, preserving every quantity of blood, taken away for inspection and examination, is absolutely necessary; for when found free from buff, no further bleeding is necessary. Horses labouring under this complaint, should always be carefully attended to; for it sometimes happens, that when the horse is supposed to be going on well and no danger suspected, that he is suddenly attacked with difficulty and quickness of breathing, with other symptoms, denoting approaching inflammation of the lungs; and if at this time a large quantity of blood be taken off, the horse will be relieved, and a dangerous inflammation of an important organ will be averted. The disease has sometimes occurred in so mild a form, as to require only a little nursing for its removal. It is, however, always proper to lay the horse up, and attend to him carefully, the disorder sometimes suddenly assuming a more serious appearance, without any visible cause. To attempt to work a horse in this complaint, however slightly you may do it, is highly improper; in several cases that have come under my notice, life has been endangered, and such debility produced, so as to render the animal useless for many months, and an incurable cough is not an unfrequent effect of such imprudence.

Having thus given a detailed account of the epidemic, which prevailed in Osmer and Gibson's time, as well as that which has occurred and fallen under my own practice and observation in this city, it may be proper to remark, that notwithstanding the apparent perplexity of the symptoms in Osmer's description, from his dividing the disease into five classes, and the difference between his and that which I have given of those which have occurred in this city, compared with those which occurred during my studies at the Veterinary College, they are really the same kind of disorder, though different in degrees; this difference may be traced in all epidemic diseases. At the same time a careful examination and comparison of them, will prove that there is no essential difference in our mode of treatment. Though the remedy he recommends as a diuretic, I certainly cannot approve of; but any thing that will increase in a moderate degree, particularly where there is no difficulty of swallowing or weakness, I think, would be highly proper. Gibson, who wrote on horses seventy years ago, describes an epidemic very similar to that which has occurred, and which he is decidedly of opinion was very infectious. He says, " about the end of the year 1732, there was a remarkable distemper of this kind among the horses in London, and several other parts of the kingdom. They were suddenly seized with a dry, vehement, and sounding cough, which shook them so violently, that some of them were ready to drop down with hard straining and want of breath. Their throats were raw and sore, and many of them had hard kernels, swelled and sore to the touch. For the first two or three days, most of them refused all manner of food and water,* and had so many other bad signs, that, when the disorder first broke out, many were afraid of a mortality coming among them; a running at the nose generally came, begun on the third day, and continued in so profuse a manner for five or six days, that some of them discharged as much, in that time, of purulent matter, as two or three pails would hold;† while the running at the nose continued, they did not feed much, and the loss of flesh was very great; but so soon as the running abated, they eat voraciously, and soon recovered their strength."

From many of Gibson's remarks, he considered this a contagious disease. Several horses have certainly been attacked in the same stable, and among those belonging to Mr. Tomlinson, on the Lancaster line, it appears that three horses out of a team, and sometimes the whole four have been attacked in succession. It is necessary here to remark, that horses subjected to the draught, have appeared to suffer more from the complaint than others, particularly where they were (as those certainly are) exposed to sudden changes of the weather. It should also be taken into consideration, that hot and ill ventilated stables, are particularly favourable, to its production as well as progress, and horses that are fed high, as all stage and post horses are, appear more susceptible of the disease than those of other work. The way in which those horses of Mr. Tomlinson's were first attacked, I will endeavour to detail in his own words as near as possible, for

* As was universally the case with all the horses attacked, belonging to Mr. Tomlinson, on the stage line to Lancaster.

† Though in no instance has the running at the nose been so profuse in any of those cases attended by me. The case of Mr. Hampton's horse, and also a horse belonging to Mr. Allen, was to be sure, uncommonly great, particularly when the nose bag was applied.

I did not myself see them till several days afterwards. The first symptoms were a loss of appetite, which began with violent shivering, a cold skin, quick pulse, and a laborious perspiration, attended with cough and a discharge from both nostrils, which began a few days after the commencement of the attack; two cases which I attended at Downingstown, had already terminated in inflamed lungs. These two horses had been attacked some days; I found them labouring under the above disease, with a strong pulse at 95, though not so strong at either the temporal or maxillary artery. I took away five quarts of blood, and administered such medicine as I expected would lower the action of the heart and arteries, and in the morning, the horses appeared much relieved, and the pulse reduced to 70. The medicine was repeated, and I did not see them till the same evening, about six o'clock—pulse reduced to about 60, when the same medicine was given in the same quantity; during the night they had ate up all their feed, and in the morning appeared more lively, and the cough much relieved from the application of a former blister, and in this convalescent state I left them, with proper directions, and under perfect hope of recovery. Of the other two horses, ten miles further, at Sadsbury, one horse was on the mend, and as I considered, out of danger; the other had terminated in glanders.

It may be necessary here to remark, that from the accounts which I received from England, in the months of May and June 1815, the weather it appears, had been extremely variable, and that cold winds and rains were more prevalent than usual. It should be observed, also, that it was a part of the year when catarrhal complaints or colds, were very common among young horses. How far the combination of those causes may have produced the late epidemic, independent of contagion I am not prepared to determine. It however appears from every inquiry which I could make among the farmers to the westward, while on my journey to see Mr. Tomlinson's horses, that the weather for the time of the year, had been very variable also, the sun through the day being very warm, and the mornings and evenings remarkably cold and frosty.

I cannot, however, conclude this subject, being one of importance, as it regards horses, without impressing on the mind of the reader, the necessity there is, in these cases, of the strictest attention on the part of the groom; for I do not know any disease excepting inflamed lungs, that requires so much diligence and nursing, as those which have now come under our notice. I also feel it my duty not to quit this subject without saying a few words respecting a servant belonging to Mr. Allen; he is a Dutchman, and I believe goes by the name of John; the faithful diligence with which this man attended two or three of Mr. Allen's horses, during the severity of the complaint under which they were suffering, command my higest praise. I cannot, at the same time, omit returing my thanks to Messrs. Allen and Carter, for their ready and cheerful compliance in all my requests, when attendance, assistance, or aid, in any shape was necessary, and to their unremitting diligence and attention, by good nursing, to which I attribute my success in the recovery of every horse.

J. CARVER,V. S.

One horse belonging to Mr. Ideler, of Arch-street, I believe, died: this case I was not called to attend.

